# Risk trajectories of complications in over one thousand newly diagnosed individuals with type 2 diabetes

**DOI:** 10.1038/s41598-022-16135-0

**Published:** 2022-07-11

**Authors:** Gudrun Höskuldsdóttir, Stefan Franzén, Katarina Eeg-Olofsson, Björn Eliasson

**Affiliations:** 1 Department of Medicine, Sahlgrenska University Hospital, 413 46 Gothenburg, Sweden; 2 Department of Molecular and Clinical Medicine, Sahlgrenska Academy, University of Gothenburg, Gothenburg, Sweden; 3 National Diabetes Register, Centre of Registers, Gothenburg, Sweden

**Keywords:** Cardiovascular diseases, Endocrine system and metabolic diseases, Kidney diseases, Metabolic disorders

## Abstract

Although the increased risk of complications of type 2 diabetes (T2D) is well known, there is still little information about the long-term development of comorbidities in relation to risk factors. The purpose of the present study was to describe the risk trajectories of T2D complications over time in an observational cohort of newly diagnosed T2D patients, as well as to evaluate the effect of common risk factors on the development of comorbidities. This national cohort study investigated individuals with T2D in the Swedish National Diabetes Register regarding prevalence of comorbidities at the time of diagnosis, and the incidence of cardiovascular disease (CVD), chronic kidney disease (CKD) and heart failure in the entire patient cohort and stratified by HbA1c levels and age at baseline. Multivariable Cox regressions were used to evaluate risk factors predicting outcomes. We included 100,878 individuals newly diagnosed with T2D between 1998 and 2012 in the study, with mean 5.5 years follow-up (max 17 years). The mean age at diagnosis was 62.6 ± SD12.5 years and 42.7% of the patients were women. Prevalent CVD was reported for 17.5% at baseline. Although the prevalence of comorbidities was generally low for individuals 50 years or younger at diagnosis, the cumulative incidence of the investigated comorbidities increased over time. Newly diagnosed CVD was the most common comorbidity. Women were shown to have a lower risk of developing comorbid conditions than men. When following the risk trajectory of comorbidities over a period of up to 15 years in individuals with type 2 diabetes, we found that all comorbidities gradually increased over time. There was no distinct time point when onset suddenly increased.

## Introduction

Type 2 diabetes (T2D) is a chronic disease caused by the progressive loss of insulin secretion by pancreatic beta cells coupled with insulin resistance, but other pathogenetic mechanisms also contribute^1^. Asymptomatic impaired glucose regulation can however precede the fulfilment of diagnostic criteria for months and even years, and consequently, low grade hyperglycemia during this period can contribute to increase in risk for micro- and macrovascular disease. Therefore, diabetes-related co-morbidities and complications, such as retinopathy and proteinuria, may well present at the time of diagnosis^2^. Retinopathy is often used as a surrogate for the presence of co-morbidities and it has been reported that 36% of newly diagnosed diabetes patients may have retinopathy^3^.

Compared to the general population, individuals with T2D have a 2–4 times higher risk of death and developing cardiovascular disease^4^. The prevalence of heart failure is also considered to be up to four times higher in individuals with T2D compared to the general population even in individuals with good glycaemic control but may well still be underdiagnosed^5,6^. In individuals with diabetes, impaired insulin signalling and decreased uptake of glucose by skeletal muscle cause hyperglycaemia and hyperlipidaemia. Despite this the contribution of glycolysis in cardiac ATP production is decreased and the heart relies on fatty acid oxidation to a greater degree^7^. Furthermore, chronic kidney disease (CKD, i.e., persistent presence of albuminuria or lower estimated glomerular filtration rate (eGFR)), occurring in up to 50% of individuals with diabetes, is also associated with increased cardiovascular risk^8^. Consequently, atherosclerotic cardiovascular disease, i.e., coronary heart disease, cerebrovascular disease or peripheral arterial disease of atherosclerotic origin, in individuals with diabetes was estimated to entail 37.3 billion US dollars per year in cardiovascular costs in the US^9^. The treatment of type 2 diabetes should therefore be characterized by multifactorial risk factor intervention, which has been clearly shown to reduce micro- and macrovascular complications and death^10,11^.

Although the increased risk of complications of type 2 diabetes is well known, there is still too little information about the long-term development of comorbidities in relation to risk factors. Accordingly, the purpose of the present study was to describe the risk trajectories of T2D complications over time in an observational cohort of newly diagnosed T2D patients, as well as to evaluate the effect of common risk factors on the development of comorbidities, using nationwide data from the Swedish National Diabetes Register (NDR).

## Methods

### Origin of data

The study is an observational retrospective cohort study based on data from NDR, which was started in 1996 and includes information the vast majority of people with diabetes mellitus in Sweden, reported by healthcare professionals^12^. In this study, the epidemiological definition of type 2 diabetes used (as presented in previous studies originating from NDR^13^) was treatment with diet only or oral hypoglycaemic drugs only, or, if onset of diabetes was at age 40 years or older, treatment with insulin only or combined with oral hypoglycaemic drugs. All patients have approved to be reported to NDR, but Swedish law does not require individual consent for inclusion in the study. All methods were performed in accordance with relevant guidelines and regulations. The study was approved by the Swedish Ethical Review Authority (DNR 56312).

### Study population and end points

All persons who were at least 18 years of age diagnosed with T2D between 1 January 1998 and 31 December 2012 and registered in NDR were included in the study. Exclusion criteria were type 1 diabetes, secondary diabetes or an indication of gestational diabetes within 8 months from the time of T2D diagnosis, or missing information about the time of onset of T2D. We used three endpoints in the study: a composite cardiovascular outcome (CVD, including myocardial infarction, stable and unstable angina pectoris and stroke), heart failure and chronic kidney disease.

### Data collection and data sources

All information was extracted by linking the various databases using all participants' unique social security numbers. Baseline information at the index date (date of registered diagnosis) or up to six months later, was retrieved from NDR. If multiple values were available from the same date, the average value was used. Diagnoses reported during the first six months after the index date were considered as previous conditions. Incidence of outcomes were assessed after 2, 5, 10, 13 and 15 years, based on hospitalizations and reported diagnoses in the medical journals according to the International Classification of Diseases, 10th revision (ICD-10) codes (presented in supplementary Table S1). Information on hospital admissions, socioeconomic variables, and dates and causes of death were retrieved from the National Patient Registry, the Longitudinal Integration Database for Health Insurance and Labor market studies until the end of 2013 and the Cause of Death register until the end of 2014, respectively^14–16^.

### Statistical analysis

Analyses were stratified according to age (18–29, 30–39, 40–49, 50–59, 60–69, 70–79, and 80 +) and baseline HbA1c (< 7.0%, 7.0–7.4%, 7.5–7.9%, ≥ 8%, or missing). Continuous variables were presented as the mean with standard deviation, and categorical variables as frequencies and proportions. Descriptive statistics were presented both overall and by strata. The prevalence of each comorbidity at diagnosis and 95% confidence intervals (CI) were also presented overall and split by strata. Standardised mean difference (SMD), a measure of distance between the group means (difference between sample means divided by pooled SD), was used to compare group characteristics, with SMD > 0.1 denoting a statistical difference. The date of diagnosis was registered as the year of initial diagnosis. Therefore, the cumulative incidence of each event was reported as a life table. The analysis for each outcome was conducted among the population at risk. Patients with prior history of the outcome of interest were excluded from analysis of that particular outcome.

Patients were followed from the index date until the outcome event, death or the end of the study. The incidence rate (IR) for each outcome was calculated for the population at risk and reported per 1,000 person-years and 95% exact Poisson CI within each stratum and after 2, 5, 10, 13, and 15 years. When the sample size in a particular stratum was small, the category was either combined with other strata or left out. Multivariable Cox regression analyses were performed for the time to complications, including all clinical variables based on ten imputed datasets (imputed multiple chained equations). R 4.0.2 was used for all statistical calculations.

### Ethics approval and consent to participate

All patients have approved to be reported to NDR, but Swedish law does not require individual consent for inclusion in the study. The study was approved by the Swedish Ethical Review Authority (DNR 56312).

## Results

### Baseline characteristics

Overall, we identified 100,878 individuals with T2D eligible for inclusion in the study (Table 1). The average follow-up time (time to censoring or death) was 5.5 years (range 1–17 years). The number of fatalities during the follow-up period was 89,542 (23.0%), and the mean time from diagnosis to death was 10.4 (range 1–27) years. The mean age of the participants was 62.2 years and 42.8% were female. The most common treatment for diabetes during the study period was diet only (53.1%) and the mean level of HbA1c at diagnosis was 7.2% (National Glycohemoglobin Standardization Program, equal to 55.0 mmol/mol). Almost 62% of the participants had treatment for hypertension and the mean systolic blood pressure was 137.5/79.6 mmHg. Overweight and obesity were common, with the mean body mass index (BMI) of the participants being 30.5 kg/m^2^. The mean level of LDL cholesterol was 3.1 µmol/L and 37.4% of the patients were treated with lipid lowering drugs. Less than a fifth of the patients smoked. Approximately 44% of the participants had a college education or higher and 53% were married. The disposable income of the participants was evenly divided between the income quartiles (22.2% quartile 1, 24.8% quartile 2, 25.1% quartile 3, and 28% quartile 4). Most of the participants originated from Sweden (82.1%).

**Table 1:** Baseline characteristics.

n	100,878
Age	62.6 (12.5)
Female	43,075 (42.8%)
BMI	30.5 (5.6)
HbA1c mmol/mol	55.0 (18.0)
HbA1c %	7.2 (1.6)
Systolic blood pressure (mmHg)	137.5 (17.4)
Diastolic blood pressure (mmHg)	79.6 (10.1)
Total cholesterol (µmol/L)	5.2 (1.2)
LDL cholesterol (µmol/L)	3.1 (1.0)
HDL cholesterol (µmol/L)	2.1 (0.5)
Macroalbuminuria	2821 (4.6%)
Microalbuminuria	5881 (12.0%)
eGFR	84.29 (25.5)
Smoking	13,875 (17.3%)
**Physical activity**	
Never	7883 (13.1%)
< once a week	7233 (12.0%)
1–2 times per week	12,308 (20.5%)
3–5 times per week	14,197 (23.6%)
Daily	18,560 (30.8%)
**Pharmaceutical treatment**	
Diabetes treatment	
Diet only	53,520 (53.1%)
Oral glucose-lowering drugs	37,497 (37.2%)
Insulin only	5979 (5.9%)
Combination	3882 (3.8%)
Anti-hypertensive treatment	57,311 (61.7%)
Lipid lowering treatment	34,715 (37.4%)
**Socioeconomic variables**	
**Highest level of education**	
Elementary school	38,105 (38.3%)
College level	43,645 (43.9%)
Upper secondary school	17,670 (17.8%)
**Marital status**	
Married	53,456 (53.0%)
Separated	17,773 (17.6%)
Single	17,932 (17.8%)
Widowed	11,636 (11.5%)
**Origin**	
Europe other than Sweden	10,305 (10.2%)
RoW	7751 (7.7%)
Sweden	82,822 (82.1%)
**Disposable income, kSEK**	**19,007.0 (20,158.0)**
**Income quartile**	
1	21,228 (22.2%)
2	23,703 (24.8%)
3	24,037 (25.1%)
4	26,767 (28.0%)
**Previous comorbidities**	
Retinopathy	2015 (12.5%)
Myocardial infarction	7882 (7.8%)
Stable angina	8225 (8.2%)
Unstable angina	3142 (3.1%)
Stroke	5135 (5.1%)
Cardiovascular disease (composite)	17,615 (17.5%)
Heart failure	4893 (4.9%)
Atrial fibrillation	7106 (7.0%)
Peripheral vascular disease	1591 (1.6%)
Amputation of lower extremity	944 (0.9%)
Chronic renal disease	911 (0.9%)
End stage renal disease	134 (0.1%)
Dementia	284 (0.3%)
**Number of previous comorbidities**	
0	76,652 (76.0%)
1	13,898 (13.8%)
2	6404 (6.3%)
3	2599 (2.6%)
4	966 (1.0%)
5	289 (0.3%)
6	59 (0.1%)
7	8 (0.0%)
8	2 (0.0%)
9	1 (0.0%)

Data reported as n (%) or * mean (SD).

*eGFR* estimated glomerular filtration rate, *RoW* rest of world, *SEK* Swedish krona.

### Stratification according to age at baseline

The participants were stratified according to age at diagnosis (supplementary Table S2). The number of patients in the seven strata were 18–29 years (n = 878), 30–39 years (n = 3717), 40–49 years (n = 12,709), 50–59 years (n = 24,301), 60–69 years (n = 31,551), 70–79 years (20,397), and over 80 years (n = 7,325). There were more men in the strata up to 70 years of age, but an even distribution of gender was seen in the stratum between 70 and 80 years. Approximately 57% of participants in the oldest age group were women. Treatment with diet only was most common in the youngest (59.9%) and oldest strata (66.3%), but other treatment options for diabetes did not follow any particular trend between the age groups. Antihypertensive treatment increased with age, while use of lipid-lowering agents increased until the age of 80 and decreased thereafter. HbA1c levels and BMI at diagnosis were inversely related with age. Levels of LDL and HDL cholesterol were similar between the groups, but the prevalence of micro- and macroalbuminuria was highest in the oldest patients (14.9% and 7.2%, respectively). Retinopathy was present in ≥ 12% of individuals over forty years of age. Between 23 and 25% of the population that was younger than sixty years of age at the time of diagnosis were registered smokers. More than half of the individuals under the age of 80 reported physical activity at least three times per week. Disposable income was highest in individuals between 40 and 60 years of age at the time of diagnosis.

### Stratification according to HbA1c levels at baseline

HbA1c levels at baseline were available for 86,967 individuals, and used to divide the dataset into four strata, i.e., < 7% (n = 55,462), 7.0–7.4% (n = 8735), 7.5–7.9% (n = 4698), and ≥ 8.0% (n = 18,072) (supplementary Table S3). There was an inverse relationship between the level of HbA1c at baseline and age, with the highest HbA1c levels noted in the youngest individuals. Treatment with diet only was most common in the group with the lowest HbA1c levels at diagnosis (66.6%) and treatment that included insulin was more common in the stratum with the highest HbA1c levels at diagnosis (11.5% and 8.2% with insulin only or a combination of oral glucose-lowering treatment and insulin in the ≥ 8% strata, respectively). Levels of systolic blood pressure, HDL, and protein in the urine were similar in all groups, but there were minor differences with regards to levels of diastolic blood pressure, LDL and eGFR (SMD > 0.1). Body mass index (between 30 and 31 kg/m^2^), the percentage of smokers, education levels, disposable income, and country of origin were similar for all strata, but there were slight differences with regards to the levels of physical activity and civil status.

### Prevalent comorbidities

Cardiovascular disease (including previous myocardial infarction, stable or unstable angina or stroke) was present in 17.5% of the population at baseline (Table 1). Almost 5% of the participants had known heart failure, however less than 1% had renal failure. Close to a fourth of the population (24%) already had at least one known comorbidity when diagnosed with diabetes. Comorbidities at baseline were uncommon (≤ 5%) in patients that were 50 years old or younger at the time of diagnosis (Supplementary Table S1).

The prevalence of cardiovascular disease was similar in the different HbA1c strata at baseline (Supplementary Table S2, as well as previous heart failure, atrial fibrillation, chronic or end-stage kidney disease, peripheral vascular disease and non-traumatic amputation of lower extremities. Dementia was uncommon in all strata (< 0.5%). The number of comorbidities present at baseline was slightly different between the HbA1c strata but did not follow any particular trend.

### Incidence of cardiovascular disease, heart failure or kidney failure

**Figure 1:**
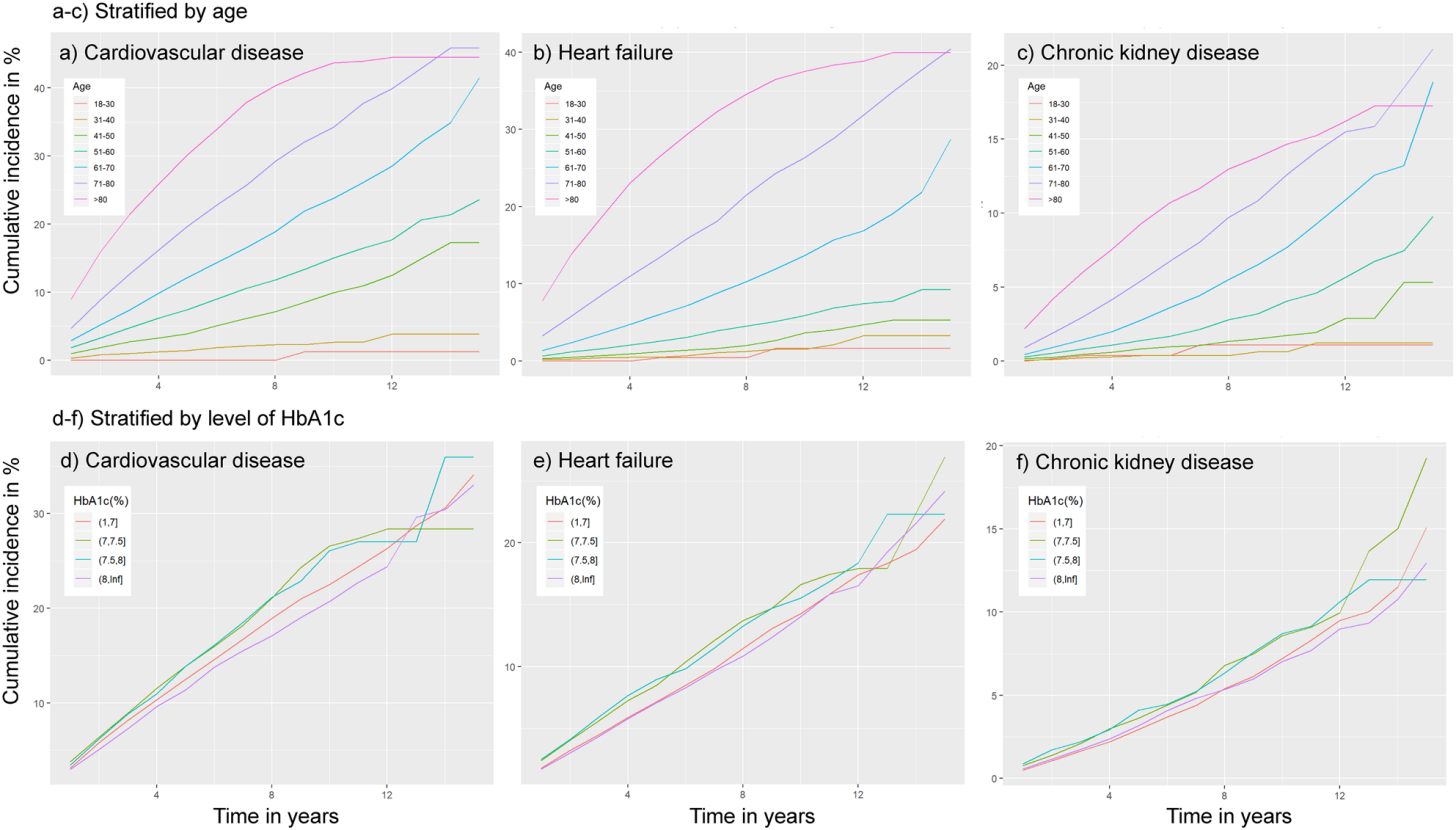
Cumulative incidence of complications. Line plots illustrating cumulative incidence (CI) (%) stratified by age (**A**–**C**) or HbA1c levels (**D**–**F**) at the time of diagnosis for cardiovascular disease (CVD), heart failure (HF), and chronic kidney disease (CKD). CI is presented on the y-axis and time from diagnosis on the x-axis (years). Note different scales on Y axes.

The incidence rates for the outcomes are given in Table 2, and the cumulative incidence of cardiovascular disease, heart failure and renal disease is given in Fig. 1, stratified by age (Fig. 1A–C) and HbA1c levels at the time of diagnosis (Fig. 1D–F). The IR for cardiovascular disease, heart failure, and renal failure in the full cohort was 15.9 (95% CI 15.5; 16.3), 9.4 (95% CI 9.1; 9.7), and 5.3 (95% CI 5.1; 5.5), respectively. Increasing age was associated with increasing IR for all outcomes. After the population was stratified according to HbA1c levels at diagnosis, the incidence rate for cardiovascular disease was highest in the group that presented with HbA1c levels between 7.0 and 7.4%. The incidence rate for heart failure was elevated but similar for the 7.0–7.4% (10.5 (95% CI 9.5; 11.6) and 7.5–7.9% (10.9 (95% CI 9.5; 12.5) strata. Renal failure was generally less common, with an incidence rate between 5.1 and 6.4 in all strata.

**Table 2:** Incidence rate of cardiovascular disease, heart failure or kidney failure.

	Cardiovascular disease	Heart failure	Kidney failure
Total population	15.9 (15.5;16.3)	9.4 (9.1;9.7)	5.3 (5.1;5.5)
**Stratified by age at diagnosis (years)**			
18–29	0.3 (0;1.5)	0.6 (0.1;2)	0.8 (0.2;2.4)
30–39	2.2 (1.6;3.1)	1 (0.6;1.6)	0.4 (0.1;0.8)
40–49	6.2 (5.5;6.9)	1.7 (1.4;2.1)	1.2 (0.9;1.5)
50–59	10.4 (9.7;11)	3.6 (3.3;4)	2.6 (2.3;2.9)
60–69	16.2 (15.4;17)	8 (7.5;8.5)	5 (4.6;5.4)
70–79	27.9 (26.6;29.2)	18.4 (17.5;19.3)	9.2 (8.6;9.9)
≥ 80	45.2 (42.2;48.3)	35.2 (33;37.6)	14.4 (13.1;15.7)
**Stratified by HbA1c (%) at diagnosis**			
< 7.0	15.8 (15.3;16.4)	9.4 (9;9.8)	5.1 (4.9;5.4)
7–7.4	18.2 (16.8;19.8)	10.5 (9.5;11.6)	6.3 (5.6;7.1)
7.5–8.0	17.5 (15.6;19.6)	10.9 (9.5;12.5)	6.4 (5.4;7.6)
≥ 8.0	15.9 (14.9;16.9)	8.7 (8;9.4)	5.3 (4.8;5.8)

Incidence rate per 1000 person years of outcomes with exact 95% Poisson CI.

### Evaluation of risk factors

Multiple Cox regressions were used to evaluate risk factors predicting outcomes (Table 3). Female sex was clearly associated with decreased risk for cardiovascular disease, heart failure, and kidney disease. Increasing levels of physical activity was associated with a similar effect, but not regarding kidney disease. Smoking was strongly linked to elevated hazard ratios (HR) for all outcomes. Glucose-lowering drug use was also associated with increased risks. Micro- and macroalbuminuria, the latter in particular but not eGFR, was associated with heart failure and, most of all, kidney failure (Table 3).

**Table 3:** Risk for comorbidity.

Variable	HR	95% CI	p
**Cardiovascular disease**			
Age	1.054	(1.053; 1.055)	< 0.001
Female	0.669	(0.657; 0.681)	< 0.001
Oral antihyperglycemic agents (OAH)	1.085	(1.064; 1.106)	< 0.001
Insulin only	1.105	(1.067; 1.145)	< 0.001
OAH and insulin	1.368	(1.314; 1.425)	< 0.001
HbA1c	1.001	(1.001; 1.002)	< 0.001
Systolic blood pressure	1.006	(1.006; 1.007)	< 0.001
Diastolic blood pressure	0.996	(0.995; 0.997)	< 0.001
Anti-hypertensive treatment	1.163	(1.141; 1.185)	< 0.001
BMI	0.999	(0.997; 1.001)	0.184
LDL	1.068	(1.059; 1.076)	< 0.001
HDL	1.042	(1.025; 1.059)	< 0.001
Lipid lowering treatment	1.050	(1.031; 1.070)	< 0.001
Macroalbuminuria	1.087	(1.049; 1.126)	< 0.001
Microalbuminuria	1.052	(1.027; 1.077)	< 0.001
eGFR	0.998	(0.998; 0.998)	< 0.001
Smoking	1.345	(1.315; 1.375)	< 0.001
**Physical activity**			
Never	1.011	(0.979; 1.044)	0.489
< once a week	0.938	(0.911; 0.965)	< 0.001
1–2 times per week	0.896	(0.870; 0.922)	< 0.001
3–5 times per week	0.928	(0.903; 0.953)	< 0.001
**Heart failure**			
Age	1.084	(1.083; 1.086)	< 0.001
Female	0.714	(0.699; 0.729)	< 0.001
Oral antihyperglycemic agents (OAH)	1.040	(1.016; 1.065)	< 0.001
Insulin only	1.356	(1.302; 1.411)	< 0.001
OAH and insulin	1.386	(1.318; 1.456)	< 0.001
HbA1c	1.003	(1.002; 1.003)	< 0.001
Systolic blood pressure	1.000	(1.000; 1.001)	0.294
Diastolic blood pressure	0.995	(0.994; 0.996)	< 0.001
Anti-hypertensive treatment	1.390	(1.355; 1.425)	< 0.001
BMI	1.032	(1.030; 1.034)	< 0.001
LDL	0.948	(0.938; 0.958)	< 0.001
HDL	0.960	(0.940; 0.980)	< 0.001
Lipid lowering treatment	1.109	(1.085; 1.134)	< 0.001
Macroalbuminuria	1.214	(1.169; 1.262)	< 0.001
Microalbuminuria	1.128	(1.097; 1.160)	< 0.001
eGFR	0.996	(0.995; 0.996)	< 0.001
Smoking	1.350	(1.311; 1.390)	< 0.001
**Physical activity**			
Never	0.861	(0.831; 0.892)	< 0.001
< once a week	0.747	(0.724; 0.772)	< 0.001
1–2 times per week	0.709	(0.686; 0.732)	< 0.001
3–5 times per week	0.741	(0.719; 0.763)	< 0.001
**Renal failure**			
Age	0.981	(0.974; 0.988)	< 0.001
Female	0.538	(0.464; 0.623)	< 0.001
Oral antihyperglycemic agents (OAH)	0.999	(0.841; 1.187)	0.991
Insulin only	4.484	(3.761; 5.346)	< 0.001
OAH and insulin	0.732	(0.464; 1.155)	0.180
HbA1c	0.999	(0.995; 1.003)	0.577
Systolic blood pressure	1.013	(1.009; 1.017)	< 0.001
Diastolic blood pressure	0.997	(0.989; 1.005)	0.449
Anti-hypertensive treatment	1.503	(1.268; 1.781)	< 0.001
BMI	0.991	(0.977; 1.005)	0.188
LDL	0.882	(0.824; 0.945)	< 0.001
HDL	1.023	(0.906; 1.155)	0.712
Lipid lowering treatment	0.707	(0.609; 0.822)	< 0.001
Macroalbuminuria	5.386	(4.618; 6.282)	< 0.001
Microalbuminuria	1.145	(0.954; 1.374)	0.145
eGFR	0.955	(0.951; 0.959)	< 0.001
Smoking	1.954	(1.666; 2.292)	< 0.001
**Physical activity**			
Never	1.262	(0.991; 1.608)	0.059
< once a week	0.988	(0.786; 1.243)	0.921
1–2 times per week	1.163	(0.933; 1.451)	0.179
3–5 times per week	1.110	(0.898; 1.372)	0.334

Results from multiple Cox regressions used to evaluate risk factors predicting outcomes.

*BMI* body mass index, HDL high density lipoprotein, *eGFR* estimated glomerular filtration rate.

## Discussion

Although the elevated risk of cardiovascular disease and premature mortality in T2D has long been established, longitudinal data on the trajectories of these and associations with risk factors, are scarce. In the present study, we followed 100,878 individuals with type 2 diabetes for up to 17 years from diagnosis and studied the prevalence of comorbidities at diagnosis, incidence of cardiovascular events, heart failure and kidney disease during the follow-up period, and evaluated the effects of different risk factors on the risk of these outcomes. There were a large number of risk factors and characteristics that were associated with an increased risk for the outcome measures studied, but some of these were particularly significant. There were, e.g., slow and steady increases in all outcomes over time, particularly for cardiovascular disease and heart failure, in people older than 60 years, but there was also a clear increase in CVD after 40 years. Our results suggest that women with T2D have a lower risk for developing severe complications than men. Physical activity seems valuable but smoking deleterious with regard to these outcomes, while albuminuria was clearly linked to heart failure and kidney disease, in particular. Glucose-lowering medications, including insulin, seemed to be associated with elevated risks, which can be explained by reversed causality as the need for treatment including insulin implies progression of the disease.

Serious diabetic complications and premature death occur less frequently nowadays, as shown in studies from the USA, England and Sweden^4,17,18^. In the present study, higher age was clearly associated with increased risk for the three main outcomes. However, a previous study shows that higher age primarily means an increased excess risk of mortality, myocardial infarction, stroke and heart failure with increasing numbers of uncontrolled cardiovascular risk factors (HbA1c, LDL cholesterol, albuminuria, smoking, blood pressure), but the persons at highest excess risk were younger persons (under 55 years of age)^11^. It seems reasonable to suggest that the higher risk for the outcome measures studied in the present study was also explained by the presence of another conditions, i.e., concomitant morbidity with one to four cardiovascular diagnoses (Supplementary table S1). The younger individuals lacked such, although their HbA1c as well as BMI were higher.

Female gender was a protective characteristic, and the proportion of women was highest among the youngest and oldest persons. Among people with diabetes, men have been noted to have a significantly increased risk of death compared to women, but have also experienced a reduction in the excess risk of death during the period 1990–2017, according to a recent US study^19^. The differences in the risk of cardiovascular disease seen between men and women have been the subject of discussion for many years, but it seems that the condition involves an increased risk of death when it occurs in women compared to men^20,21^. Diabetic cardiomyopathy, independent of coronary heart disease or hypertension, has also been shown to be more prevalent in women with diabetes than men with diabetes^7^.

Heart failure occurred less frequently than cardiovascular disease but similar risk factors predicted both outcomes. Severe kidney disease was also predicted by many risk factors, but most clearly by insulin monotherapy, smoking and macroalbuminuria. The strong link to antihypertensive therapy was expected due to its crucial importance in the treatment of diabetic kidney disease. Smoking has long been considered an important risk factor for chronic kidney disease, through several mechanisms^22^.

This study includes over 100,000 individuals with type 2 diabetes that were followed from diagnosis up to 17 years. This cohort should be representative, as NDR is nationwide and contains information about the vast majority of people with T2D in Sweden (approximately 90%), and the criteria for participation in the study were broad. The registers used in the study, as well as the principal methods used, have previously been described in detail^11,15^. However, it is an observational study and we are dependent on the accuracy of register data, i.e., the diagnostic codes reported in clinical everyday life. By using ICD-10 codes after hospitalisations to retrieve information on comorbidities, we lose information on events not requiring treatment in hospital. The ICD-10 codes for heart failure also provide limited information on subtypes of heart failure regarding ejection fraction. We have not examined the effects long-term levels of risk factors or use of medications, and one additional limitation of the study is the inclusion period, as treatment recommendations in early T2D and pharmaceutical options have changed considerably during the last 10 years, especially with regards to cardiovascular and renal conditions. The recommendations for treatment concomitant heart failure and T2D have also recently been updated^23^. Not only have several new drugs been introduced, but the recommendations even regarding metformin use and kidney function have changed, allowing metformin to be used even at lower eGFR^24^. The treatment options for concomitant obesity and T2D have also changed with more emphasis being placed on bariatric surgery, lifestyle education, and even the addition of new pharmaceutical drugs, which are starting to be increasingly used after the study period^25^. However, even with more focus on lifestyle, recent studies have implied that almost 50% of individuals with T2D diabetes do not meet the recommended minimum for daily levels of physical activity and the majority spending their time sleeping or involved in sedentary activities^26^. The time since that has passed since end of follow-up might also been seen as a limitation. However, this study describes the natural course of T2D before the addition of SGLT2 inhibitors and GLP-1 receptor agonists which is of value.

The study was intended to describe the prevalence of severe cardiovascular disease, heart failure or severe kidney disease among people with newly onset type 2 diabetes, and to study the significance of a large number of established risk factors. Those that stand out as particularly significant are smoking and albuminuria, while female gender and physical activity are protective. There are also strong associations with drug treatment that is glucose, blood pressure and lipid lowering, advocating reverse causality because the respective treated risk factors do not fall out in our analysis. It is certainly possible that the risk factors may have different meanings among people of different ages and with different risk factor burdens, but good treatment of the risk factors can mean that the risk of complications remains low even at an older age^11^.

## Supplementary Information

Supplementary Information.

## Data Availability

The datasets used and/or analysed during the current study available from the corresponding author on reasonable request.

## Abbreviations

BMI: Body mass index
CKD: Chronic kidney disease
CVD: Cardiovascular disease
DBP: Diastolic blood pressure
eGFR: Estimated glomerular filtration rate
HbA1c: Glycated hemoglobin
HDL: High density lipoprotein
LDL: Low density lipoprotein
NDR: Swedish national diabetes register
SBP: Systolic blood pressure
SMD: Standardised mean difference
T2D: Type 2 diabetes
