# Supplementary material for: Risk trajectories of complications in over one thousand newly diagnosed individuals with type 2 diabetes
Source: Sci Rep. 2022 Jul 11;12:11784. doi: 10.1038/s41598-022-16135-0 (PMC9276720; doi:10.1038/s41598-022-16135-0)
Supplement: Supplementary file 1 — Supplementary Information. [file 41598_2022_16135_MOESM1_ESM.docx]

**Supplementary table 1.** ICD 9 and 10 codes retrieved from medical journals

|  | **ICD9** | **ICD10** |
| --- | --- | --- |
| **Myocardial infarction** | 410 | I21 |
| **Stable angina** | 413 | I201, I208, I209, I20.1, I20.9, I20.8 |
| **Unstable angina** | 4111, 4118, | I20.0 |
| **Stroke** | 431, 432, 433, 434, 436 | I61, I62, I63, I64 |
| **Heart failure** | 428 | I50 |
| **Atrial fibrillation** | 427D | I48 |
| **Peripheral vascular disease** | 250.7, 440.2, 440.20, 440.21, 440.22, 440.23, 440.24, 440.29, 443.1, 443.81, 443.9 | I70, I72, I73, I74, I77,  E115, E135, E145 |
| **Lower extremity amputation (non-traumatic)** | 84.1, 84.0, 84.12, 84.15, 84.17, 84.19 | NHQ09, NHQ11, NHQ12, NHQ13, NHQ14, NHQ16, NHQ17,  NHQ99, NGQ09, NGQ19, NGQ99, NFQ19, NFQ99 |
| **Chronic kidney disease** | 584, 585, 586 | N17, N18, N19  E11.2, E13.2, E14.2 |
| **End-stage renal disease** | V45B, V56 | Z940, Z491, Z492, Z992 |
| **Dementia** |  | F00, F01, F02, F03, G31, G30 |

**Supplementary table 2**. Baseline characteristics stratified by age at diagnosis

|  | **0-29** | **30-39** | **40-49** | **50-59** | **60-69** | **70-79** | **>80** | **p** | **SMD** |
| --- | --- | --- | --- | --- | --- | --- | --- | --- | --- |
| N | 878 | 3717 | 12709 | 24301 | 31551 | 20397 | 7325 |  |  |
| Age in years | 25.6 (3.6) | 36.8 (2.7) | 46.2 (2.8) | 56.0 (2.86) | 65.3 (2.8) | 75.0 (2.8) | 84.4 (3.1) | <0.001 | 8.6 |
|  |  |  |  |  |  |  |  |  |  |
| Female | 428 (48.7) | 1517 (40.8) | 4643 (36.5) | 9251 (38.1) | 13017 (41.3) | 10033 (49.2) | 4186 (57.1) | <0.001 | 0.18 |
| Diabetes treatment |  |  |  |  |  |  |  | <0.001 | 0.36 |
| Diet only | 526 (60.0) | 1748 (47.0) | 5238 (41.2) | 11710 (48.2) | 17147 (54.3) | 12296 (60.3) | 4855 (66.3) |  |  |
| OAH | 352 (40.1) | 1809 (48.7) | 5501 (43.3) | 9894 (40.7) | 11593 (36.7) | 6547 (32.1) | 1801 (24.6) |  |  |
| Insulin | 0 (0.0) | 107 (2.9) | 1203 (9.5) | 1555 (6.4) | 1615 (5.1) | 996 (4.9) | 503 (6.9) |  |  |
| OAH and insulin | 0 (0.0) | 53 (1.4) | 767 (6.0) | 1142 (4.7) | 1196 (3.8) | 558 (2.7) | 166 (2.3) |  |  |
| Hba1c (mmol/mol) | 61.1 (23.7) | 58.7 (20.4) | 58.7 (20.2) | 56.6 (19.3) | 53.9 (17.2) | 52.4 (15.4) | 53.2 (15.0) | <0.001 | 0.21 |
| Hba1c(%) | 7.7 (2.2) | 7.5 (1.9) | 7.5 (1.9) | 7.3 (1.8) | 7.1 (1.6) | 6.9 (1.4) | 7.0 (1.4) | <0.001 | 0.21 |
| SBP(mmHg) | 125.1 (13.9) | 127.9 (15.5) | 131.6 (16.9) | 136.3 (16.6) | 139.1 (17.0) | 140.6 (17.8) | 141.6 (18.9) | <0.001 | 0.48 |
| DBP(mmHg) | 77.4 (9.9) | 80.3 (10.6) | 81.9 (10.3) | 81.9 (9.8) | 79.8 (9.4) | 77.0 (9.8) | 74.9 (10.2) | <0.001 | 0.32 |
| Anti-hypertensive treatment | 57 (7.1) | 651 (19.4) | 4240 (36.8) | 12325 (55.1) | 20114 (68.9) | 14526 (76.7) | 5398 (79.8) | <0.001 | 0.86 |
| BMI (kg/m2) | 34.7 (9.0) | 33.80 (7.3) | 32.34 (6.4) | 31.01 (5.5) | 30.27 (5.2) | 29.07 (4.8) | 27.56 (4.4) | <0.001 | 0.51 |
| Total cholesterol (umol/L) | 5.0 (1.2) | 5.2 (1.2) | 5.4 (1.2) | 5.4 (1.2) | 5.2 (1.2) | 5.1 (1.1) | 5.1 (1.1) | <0.001 | 0.16 |
| LDL (umol/L) | 3.0 (0.9) | 3.2 (0.9) | 3.2 (1.0) | 3.23 (1.0) | 3.1 (1.0) | 3.0 (1.0) | 3.0 (1.0) | <0.001 | 0.16 |
| HDL (umol/) | 1.9 (0.5) | 2.0 (0.5) | 2.1 (0.5) | 2.1 (0.5) | 2.1 (0.5) | 2.1 (0.4) | 2.1 (0.4) | <0.001 | 0.17 |
| Lipid lowering treatment | 38 (4.8) | 457 (13.5) | 2861 (24.6) | 7873 (35.1) | 12884 (44.2) | 8454 (44.9) | 2148 (32.4) | <0.001 | 0.45 |
| Macro albuminuria | 14 (3.2) | 87 (3.9) | 317 (4.1) | 591 (3.9) | 786 (4.1) | 724 (5.8) | 302 (7.2) | <0.001 | 0.07 |
| Micro albuminuria | 37 (10.2) | 213 (11.4) | 742 (11.5) | 1358 (11.0) | 1817 (11.6) | 1278 (13.3) | 436 (14.9) | <0.001 | 0.06 |
| eGFR | 118.27 (28.67) | 107.29 (27.42) | 98.98 (29.67) | 91.11 (22.77) | 82.91 (21.41) | 72.93 (21.45) | 63.68 (18.76) | <0.001 | 0.97 |
| Retinopathy | 4 (3.7) | 45 (8.5) | 259 (11.9) | 504 (12.6) | 682 (13.2) | 414 (12.9) | 107 (11.8) | 0.003 | 0.13 |
| Smoking | 131 (23.8) | 649 (23.1) | 2526 (25.3) | 4680 (24.0) | 4229 (16.8) | 1446 (8.8) | 214 (3.7) | <0.001 | 0.28 |
| **Physical activity** |  |  |  |  |  |  |  | <0.001 | 0.20 |
| Never | 44 (11.3) | 270 (13.2) | 939 (12.7) | 1659 (11.6) | 2144 (11.1) | 1775 (14.2) | 1052 (24.7) |  |  |
| < once a week | 58 (14.8) | 296 (14.5) | 975 (13.2) | 1797 (12.6) | 2104 (10.9) | 1373 (11.0) | 630 (14.8) |  |  |
| 1-2 times per week | 84 (21.5) | 447 (21.9) | 1630 (22.1) | 3133 (21.9) | 3779 (19.5) | 2387 (19.2) | 848 (19.9) |  |  |
| 3-5 times per week | 101 (25.8) | 514 (25.2) | 1832 (24.8) | 3600 (25.2) | 4678 (24.2) | 2743 (22.0) | 729 (17.1) |  |  |
| Daily | 104 (26.6) | 513 (25.1) | 2005 (27.2) | 4104 (28.7) | 6647 (34.3) | 4185 (33.6) | 1002 (23.5) |  |  |
|  |  |  |  |  |  |  |  |  |  |
| **Highest education** |  |  |  |  |  |  |  | <0.001 | 0.429 |
| Elementary school | 217 (25.9) | 807 (22.1) | 2738 (21.7) | 6914 (28.7) | 12027 (38.6) | 10825 (54.1) | 4577 (64.6) |  |  |
| College level | 449 (53.5) | 2010 (55.1) | 7131 (56.6) | 11978 (49.6) | 13561 (43.6) | 6665 (33.3) | 1851 (26.1) |  |  |
| Upper secondary school | 173 (20.6) | 834 (22.8) | 2725 (21.6) | 5234 (21.7) | 5533 (17.8) | 2509 (12.5) | 662 (9.3) |  |  |
| **Marital status** |  |  |  |  |  |  |  | <0.001 | 1.02 |
| Married | 176 (20.1) | 1600 (43.1) | 5852 (46.1) | 13218 (54.4) | 18691 (59.3) | 11138 (54.6) | 2781 (38.0) |  |  |
| Separated | 30 (3.4) | 401 (10.8) | 2153 (17.0) | 5066 (20.9) | 6424 (20.4) | 3050 (15.0) | 649 (8.9) |  |  |
| Single | 670 (76.4) | 1695 (45.7) | 4579 (36.1) | 5405 (22.3) | 3819 (12.1) | 1371 (6.7) | 393 (5.4) |  |  |
| Widowed | 1 (0.1) | 17 (0.5) | 113 (0.9) | 596 (2.5) | 2590 (8.2) | 4828 (23.7) | 3491 (47.7) |  |  |
| **Origin** |  |  |  |  |  |  |  | <0.001 | 0.417 |
| Europe except Sweden | 49 (5.6) | 289 (7.8) | 1286 (10.1) | 2678 (11.0) | 3377 (10.7) | 2142 (10.5) | 484 (6.6) |  |  |
| RoW | 194 (22.1) | 996 (26.8) | 2339 (18.4) | 2455 (10.1) | 1161 (3.7) | 480 (2.4) | 126 (1.7) |  |  |
| Sweden | 635 (72.3) | 2432 (65.4) | 9084 (71.5) | 19168 (78.9) | 27013 (85.6) | 17775 (87.1) | 6715 (91.7) |  |  |
| Disposable income | 1396.3 (835.9) | 1955.2 (1150.9) | 2124.8 (1611.4) | 2162.3 (1759.6) | 1965.2 (2366.0) | 1529.1 (2112.1) | 1434.2 (1711.2) | <0.001 | 0.25 |
| **Disposable income quartile** |  |  |  |  |  |  |  | <0.001 | 0.57 |
| 1 | 341 (40.9) | 651 (18.5) | 1893 (15.7) | 3767 (16.4) | 6234 (20.6) | 5941 (30.8) | 2401 (34.9) |  |  |
| 2 | 163 (19.6) | 584 (16.6) | 1773 (14.7) | 3584 (15.6) | 7580 (25.1) | 7248 (37.6) | 2771 (40.3) |  |  |
| 3 | 192 (23.0) | 975 (27.8) | 3245 (26.9) | 6018 (26.3) | 8028 (26.5) | 4335 (22.5) | 1244 (18.1) |  |  |
| 4 | 137 (16.4) | 1301 (37.1) | 5149 (42.7) | 9555 (41.7) | 8412 (27.8) | 1753 (9.1) | 460 (6.7) |  |  |
| **Previous disease** |  |  |  |  |  |  |  |  |  |
| Myocardial infarction | 0 (0.0) | 20 (0.5) | 309 (2.4) | 1246 (5.1) | 2738 (8.7) | 2427 (11.9) | 1142 (15.6) | <0.001 | 0.31 |
| Stable angina | 0 (0.0) | 11 (0.3) | 211 (1.7) | 1073 (4.4) | 2703 (8.6) | 2798 (13.7) | 1429 (19.5) | <0.001 | 0.36 |
| Unstable angina | 0 (0.0) | 5 (0.1) | 112 (0.9) | 508 (2.1) | 1134 (3.6) | 993 (4.9) | 390 (5.3) | <0.001 | 0.18 |
| Stroke | 2 (0.2) | 17 (0.5) | 164 (1.3) | 640 (2.6) | 1642 (5.2) | 1775 (8.7) | 895 (12.2) | <0.001 | 0.26 |
| Cardiovascular disease (composite) | 2 (0.2) | 44 (1.2) | 635 (5.0) | 2623 (10.8) | 5985 (19.0) | 5625 (27.6) | 2701 (36.9) | <0.001 | 0.52 |
| Heart failure | 2 (0.2) | 16 (0.4) | 133 (1.0) | 532 (2.2) | 1329 (4.2) | 1709 (8.4) | 1172 (16.0) | <0.001 | 0.28 |
| Atrial fibrillation | 0 (0.0) | 11 (0.3) | 130 (1.0) | 635 (2.6) | 1987 (6.3) | 2720 (13.3) | 1623 (22.2) | <0.001 | 0.37 |
| Peripheral vascular disease | 1 (0.1) | 2 (0.1) | 58 (0.5) | 227 (0.9) | 497 (1.6) | 555 (2.7) | 251 (3.4) | <0.001 | 0.14 |
| Amputation of lower extremity (non-traumatic) | 1 (0.1) | 1 (0.0) | 17 (0.1) | 84 (0.3) | 221 (0.7) | 380 (1.9) | 240 (3.3) | <0.001 | 0.13 |
| Chronic renal failure | 3 (0.3) | 16 (0.4) | 58 (0.5) | 142 (0.6) | 238 (0.8) | 287 (1.4) | 167 (2.3) | <0.001 | 0.07 |
| End stage renal failure | 0 (0.0) | 6 (0.2) | 18 (0.1) | 33 (0.1) | 50 (0.2) | 24 (0.1) | 3 (0.0) | 0.233 | 0.03 |
| Dementia | 0 (0.0) | 1 (0.0) | 2 (0.0) | 10 (0.0) | 45 (0.1) | 116 (0.6) | 110 (1.5) | <0.001 | 0.08 |
| **Number of comorbidities (%)** |  |  |  |  |  |  |  | <0.001 | 0.66 |
| 0 | 869 (99.0) | 3634 (97.8) | 11811 (92.9) | 20767 (85.5) | 23590 (74.8) | 12506 (61.3) | 3475 (47.4) |  |  |
| 1 | 9 (1.0) | 62 (1.7) | 656 (5.2) | 2378 (9.8) | 4825 (15.3) | 4243 (20.8) | 1725 (23.5) |  |  |
| 2 | 0 (0.0) | 20 (0.5) | 185 (1.5) | 821 (3.4) | 2072 (6.6) | 2161 (10.6) | 1145 (15.6) |  |  |
| 3 | 0 (0.0) | 0 (0.0) | 43 (0.3) | 248 (1.0) | 749 (2.4) | 932 (4.6) | 627 (8.6) |  |  |
| 4 | 0 (0.0) | 1 (0.0) | 13 (0.1) | 71 (0.3) | 228 (0.7) | 393 (1.9) | 260 (3.5) |  |  |
| 5 | 0 (0.0) | 0 (0.0) | 1 (0.0) | 14 (0.1) | 71 (0.2) | 128 (0.6) | 75 (1.0) |  |  |
| 6 | 0 (0.0) | 0 (0.0) | 0 (0.0) | 2 (0.0) | 14 (0.0) | 28 (0.1) | 15 (0.2) |  |  |
| 7 | 0 (0.0) | 0 (0.0) | 0 (0.0) | 0 (0.0) | 0 (0.0) | 5 (0.0) | 3 (0.0) |  |  |
| 8 | 0 (0.0) | 0 (0.0) | 0 (0.0) | 0 (0.0) | 1 (0.0) | 1 (0.0) | 0 (0.0) |  |  |
| 9 | 0 (0.0) | 0 (0.0) | 0 (0.0) | 0 (0.0) | 1 (0.0) | 0 (0.0) | 0 (0.0) |  |  |

**Legend Supplementary table 2:** Data reported as n (%) or * mean (SD), **SMD:** Standardized Mean Difference, **OAH:** Oral antihyperglycemic agent**, SBP:** Systolic blood pressure, **DBP**: Diastolic blood pressure, **BMI:** Body Mass Index, **HDL:** High Density Lipoprotein, **LDL:** Low Density Lipoprotein, **eGFR:** Estimated Glomerular Filtration Rate, **RoW:** Rest of Worl

**Supplementary table 3.** Baseline characteristics stratified by HbA1c at diagnosis

|  | **<7** | **7-7.4** | **7.5-8.0** | **> 8** | **p** | **SMD** |
| --- | --- | --- | --- | --- | --- | --- |
| n | 55462 | 8735 | 4698 | 18072 |  |  |
| Age (years) * | 63.8 (12.1) | 63.2 (12.6) | 62.2 (12.8) | 59.6 (12.82) | <0.001 | 0.18 |
| Female | 24963 (45.0) | 3724 (42.6) | 1967 (41.9) | 6641 (36.7) | <0.001 | 0.09 |
| Diabetes treatment |  |  |  |  | <0.001 | 0.53 |
| Diet only | 36930 (66.6) | 4005 (45.9) | 1669 (35.5) | 4053 (22.4) |  |  |
| OAH | 15270 (27.5) | 3908 (44.7) | 2408 (51.3) | 10461 (57.9) |  |  |
| Insulin | 2095 (3.8) | 513 (5.9) | 400 (8.5) | 2077 (11.5) |  |  |
| OAH and insulin | 1167 (2.1) | 309 (3.5) | 221 (4.7) | 1481 (8.2) |  |  |
| Hba1c (mmol/mol) * | 44.97 (5.01) | 55.83 (1.40) | 60.66 (1.5) | 84.00 (17.8) | <0.001 | 2.94 |
| Hba1c (%) * | 6.26 (0.46) | 7.26 (0.13) | 7.70 (0.1) | 9.84 (1.6) | <0.001 | 2.94 |
| SBP(mmHg) * | 137.16 (17.02) | 138.28 (17.68) | 137.42 (17.3) | 138.02 (18.3) | <0.001 | 0.04 |
| DBP(mmHg * | 78.89 (9.81) | 79.80 (10.25) | 80.20 (10.2) | 81.40 (10.4) | <0.001 | 0.13 |
| Anti-hypertensive treatment | 33862 (65.8) | 4981 (62.2) | 2467 (57.7) | 8030 (50.0) | <0.001 | 0.18 |
| BMI (kg/m2) * | 30.22 (5.48) | 30.94 (5.72) | 30.99 (5.9) | 30.78 (5.9) | <0.001 | 0.07 |
| Total cholesterol (umol/L) * | 5.13 (1.1) | 5.32 (1.2) | 5.34 (1.2) | 5.58 (1.3) | <0.001 | 0.19 |
| LDL (umol/L) * | 3.05 (1.0) | 3.18 (1.0) | 3.18 (1.0) | 3.33 (1.1) | <0.001 | 0.14 |
| HDL (umol/) * | 2.06 (0.5) | 2.12 (0.5) | 2.12 (0.5) | 2.14 (0.5) | <0.001 | 0.08 |
| Lipid lowering treatment | 20565 (40.0) | 2887 (36.1) | 1454 (34.0) | 4523 (27.9) | <0.001 | 0.14 |
| Macro albuminuria | 1403 (3.7) | 322 (5.6) | 176 (5.8) | 718 (6.5) | <0.001 | 0.07 |
| Micro albuminuria | 3107 (10.1) | 529 (11.6) | 286 (11.8) | 1211 (14.0) | <0.001 | 0.06 |
| eGFR * | 81.41 (24.4) | 83.90 (25.1) | 85.08 (24.41) | 92.96 (27.41) | <0.001 | 0.23 |
| Retinopathy | 1227 (11.3) | 206 (13.7) | 132 (17.2) | 346 (16.1) | <0.001 | 0.10 |
| Smoking | 7237 (15.0) | 1261 (16.9) | 717 (17.9) | 2888 (19.5) | <0.001 | 0.07 |
| **Physical activity** |  |  |  |  | **<0.001** | **0.11** |
| Never | 4317 (11.5) | 834 (14.6) | 460 (15.2) | 1810 (16.0) |  |  |
| < once a week | 4188 (11.1) | 760 (13.3) | 404 (13.3) | 1623 (14.4) |  |  |
| 1-2 times per week | 7553 (20.1) | 1266 (22.1) | 622 (20.5) | 2304 (20.4) |  |  |
| 3-5 times per week | 9342 (24.8) | 1273 (22.3) | 659 (21.8) | 2351 (20.8) |  |  |
| Daily | 12198 (32.4) | 1586 (27.7) | 884 (29.2) | 3217 (28.5) |  |  |
| Disposable income * | 1887.7 (1788.5) | 1848.9 (1741.1) | 1866.5 (1699.6) | 1928.0 (2922.1) | 0.017 | 0.02 |
| Highest education |  |  |  |  | <0.001 | 0.04 |
| Elementary school | 21419 (39.1) | 3440 (40.1) | 1807 (39.2) | 6474 (36.4) |  |  |
| College level | 23781 (43.4) | 3689 (43.0) | 2029 (44.0) | 8082 (45.4) |  |  |
| Upper secondary school | 9567 (17.5) | 1453 (16.9) | 774 (16.8) | 3227 (18.1) |  |  |
| Marital status |  |  |  |  | <0.001 | 0.13 |
| Married | 30563 (55.1) | 4495 (51.5) | 2396 (51.0) | 8766 (48.6) |  |  |
| Separated | 9337 (16.8) | 1581 (18.1) | 812 (17.3) | 3419 (18.9) |  |  |
| Single | 8642 (15.6) | 1555 (17.8) | 930 (19.8) | 4214 (23.3) |  |  |
| Widowed | 6883 (12.4) | 1096 (12.6) | 556 (11.8) | 1651 (9.1) |  |  |
| Origin |  |  |  |  | <0.001 | 0.07 |
| Europe except Sweden | 5402 (9.7) | 862 (9.9) | 477 (10.2) | 1865 (10.3) |  |  |
| RoW | 3462 (6.2) | 679 (7.8) | 396 (8.4) | 1793 (9.9) |  |  |
| Sweden | 46598 (84.0) | 7194 (82.4) | 3825 (81.4) | 14414 (79.8) |  |  |
| Income quartile |  |  |  |  | <0.001 | 0.06 |
| 1 | 11744 (22.3) | 2002 (24.3) | 1010 (22.9) | 3731 (21.8) |  |  |
| 2 | 13342 (25.4) | 2070 (25.1) | 1109 (25.1) | 3986 (23.3) |  |  |
| 3 | 13386 (25.5) | 1955 (23.7) | 1126 (25.5) | 4195 (24.5) |  |  |
| 4 | 14080 (26.8) | 2224 (27.0) | 1170 (26.5) | 5227 (30.5) |  |  |
| **Previous disease** |  |  |  |  |  |  |
| Myocardial infarction | 4832 (8.7) | 702 (8.0) | 324 (6.9) | 939 (5.2) | <0.001 | 0.08 |
| Stable angina | 5037 (9.1) | 758 (8.7) | 367 (7.8) | 992 (5.5) | <0.001 | 0.08 |
| Unstable angina | 1965 (3.5) | 263 (3.0) | 127 (2.7) | 345 (1.9) | <0.001 | 0.05 |
| Stroke | 3004 (5.4) | 470 (5.4) | 258 (5.5) | 681 (3.8) | <0.001 | 0.04 |
| Cerebrovascular disease composite | 10721 (19.3) | 1581 (18.1) | 779 (16.6) | 2161 (12.0) | <0.001 | 0.11 |
| Heart failure | 2622 (4.7) | 532 (6.1) | 271 (5.8) | 801 (4.4) | <0.001 | 0.05 |
| Atrial fibrillation | 4037 (7.3) | 716 (8.2) | 374 (8.0) | 1023 (5.7) | <0.001 | 0.05 |
| Peripheral vascular disease | 889 (1.6) | 152 (1.7) | 80 (1.7) | 247 (1.4) | 0.061 | 0.02 |
| Amputation of lower extremity | 550 (1.0) | 96 (1.1) | 56 (1.2) | 143 (0.8) | 0.017 | 0.02 |
| Chronic renal failure | 487 (0.9) | 89 (1.0) | 53 (1.1) | 129 (0.7) | 0.012 | 0.02 |
| End stage renal failure | 68 (0.1) | 13 (0.1) | 11 (0.2) | 15 (0.1) | 0.056 | 0.02 |
| Dementia | 146 (0.3) | 35 (0.4) | 15 (0.3) | 48 (0.3) | 0.139 | 0.01 |
| **Number of comorbidities** |  |  |  |  | NaN | 0.11 |
| 0 | 41143 (74.2) | 6527 (74.7) | 3550 (75.6) | 14819 (82.0) |  |  |
| 1 | 8286 (14.9) | 1190 (13.6) | 650 (13.8) | 1882 (10.4) |  |  |
| 2 | 3755 (6.8) | 613 (7.0) | 296 (6.3) | 865 (4.8) |  |  |
| 3 | 1525 (2.7) | 260 (3.0) | 133 (2.8) | 333 (1.8) |  |  |
| 4 | 547 (1.0) | 108 (1.2) | 52 (1.1) | 123 (0.7) |  |  |
| 5 | 164 (0.3) | 29 (0.3) | 15 (0.3) | 41 (0.2) |  |  |
| 6 | 36 (0.1) | 5 (0.1) | 2 (0.0) | 8 (0.0) |  |  |
| 7 | 6 (0.0) | 1 (0.0) | 0 (0.0) | 1 (0.0) |  |  |
| 8 | 0 (0.0) | 2 (0.0) | 0 (0.0) | 0 (0.0) |  |  |
| 9 | 0 (0.0) | 0 (0.0) | 0 (0.0) | 0 (0.0) |  |  |

**Legend Supplementary table 3**: Data reported as n (%) or * mean (SD), **SMD:** Standardized Mean Difference, **OAH:** Oral antihyperglycemic agent, **SBP:** Systolic blood pressure, **DBP**: Diastolic blood pressure **BMI:** Body Mass Index, **HDL:** High Density Lipoprotein, **LDL:** Low Density Lipoprotein, **eGFR:** Estimated Glomerular Filtration Rate, **RoW:** Rest of World
